# Development of ankle and knee range of motion after isolated gastrocsoleus lengthening in children with cerebral palsy: a register-based longitudinal cohort study

**DOI:** 10.2340/17453674.2025.43387

**Published:** 2025-04-17

**Authors:** Olof LINDÉN, Henrik LAUGE-PEDERSEN, Gunnar HÄGGLUND, Philippe WAGNER

**Affiliations:** 1 Department of Clinical Sciences, Lund University, Lund; Department of Orthopedics, Skane University Hospital, Lund; 2 Center for Clinical Research, Uppsala University, Region Västmanland, Västerås, Sweden

## Abstract

**Background and purpose:**

Outcome after gastrocsoleus lengthening in cerebral palsy (CP) is reported to be influenced by type of lengthening, age, CP subtype, and preoperative range of motion (ROM). We examined the development of ankle and knee ROM after 3 types of isolated gastrocsoleus lengthening.

**Methods:**

This is a register-based longitudinal cohort study based on data from the Swedish Cerebral Palsy follow Up Program, of children born 2000–2011 who underwent isolated gastrocsoleus lengthening. ROM development was analyzed using mixed-effects modeling. Event limits were defined as ankle ROM ≤ 0° or ≥ 20° and knee extension deficit ≤ –10° and described in Kaplan–Meier curves and Cox regression analyses. The study protocol was published at clinicaltrials.gov.

**Results:**

184 children were included. The mean differences in ankle ROM 10 years postoperatively between open tendo Achilles lengthening (OTAL) and percutaneous tendo Achilles lengthening (PTAL) was –2.3° (95% confidence interval [CI] –7.4 to 2.7), and between gastrocnemius lengthening (GCL) and PTAL –4.4° (CI –10.4 to 1.5). The adjusted hazard ratio (aHR), adjusted for baseline ROM, Gross Motor Function Classification System level, and CP subtype, comparing ankle event rates between OTAL and PTAL was 2.5 (CI 1.1–5.7). GCL was also associated with a higher event rate compared with PTAL, aHR 2.0 (CI 0.85–4.6). The adjusted mean difference in knee ROM at 10 years between OTAL and PTAL was 5.1° (CI 0.4–9.8), and between GCL and PTAL 1.9° (CI –3.6 to 7.6). Comparing event rates for the knee yielded uncertain results.

**Conclusion:**

PTAL appears at least as effective as OTAL and GCL for favorable ankle and knee ROM development in children with CP.

Equinus foot position is the most prevalent deformity among children with cerebral palsy (CP) [1]. Various techniques for lengthening the gastrocsoleus complex have been described [2-5]. Surgical approaches typically target 1 of 3 anatomical zones: the gastrocnemius muscular level, its musculotendinous junction, or the Achilles tendon. In our study, surgeries performed at the muscle or musculotendinous junction are referred to as gastrocnemius lengthening (GCL). Tendo Achilles lengthenings (TAL) are divided into open TAL (OTAL) and percutaneous TAL (PTAL).

Lengthening of the gastrocsoleus complex has been associated with risk of recurrent equinus, or overlengthening sometimes leading to crouch [6,7]. A higher rate of recurrent equinus has been reported in children with unilateral spastic CP (USCP) and a higher risk of crouch and calcaneal gait in children with bilateral spastic CP (BSCP), especially after lengthening at the tendinous level [6,8,9]. In some studies, early surgery is described as a risk factor for recurrent equinus [10,11]. It has also been reported that outcome varies related to preoperative ankle ROM, more severe equinus being more prone to recurrence [12,13].

Thus the area of gastrocsoleus lengthening is not conclusive, which is why we found it of importance to report from a nationwide register.

The aim of our study was to analyze the development ankle and knee range of motion (ROM) after isolated PTAL, OTAL, and GCL.

## Methods

### Study design

This is a longitudinal register based study from the Swedish Surveillance Program for Cerebral Palsy (CPUP) registry that covers > 95% of children with CP in Sweden [14].

The study is reported according to STROBE guidelines for observational studies.

### Population

In Sweden, children with CP are followed up by multidisciplinary habilitation units, with physiotherapists conducting regular clinical assessments annually or biannually depending on age and GMFCS level (http://www.cpup.se). In CPUP, the CP diagnosis is confirmed after the age of 4 by a neuropediatrician [15,16].

### Treatment

Surgeries on children with CP are registered in CPUP, and all children born 2000–2011 who had an isolated gastrocsoleus-complex lengthening performed between 2000 and 2014 were included. The type and level of surgery were validated from surgical records for each child. Treated children were then stratified into 3 groups depending on the type and level of surgery: i.e., GCL (mainly Baker, Vulpius, and Baumann procedures), OTAL, or PTAL. The Baumann procedure [2] targets the gastrocnemius at the muscular level while Baker [17] and Vulpius procedures lengthen the gastrocnemius aponeurosis including the underlying soleus fascia [5]. Surgery at the tendinous level is performed with open Z-lengthening percutaneously at 2 or 3 levels. The final inclusion criterion was surgery performed before December 31, 2014. Children were followed up to their last assessment or the end of the study in 2021.

### Outcomes

We analyzed the effects of gastrocsoleus-complex lengthening in children with CP. We compared ankle and knee ROM development among different types and levels of gastrocsoleus lengthening. We also evaluated the associations between treatment outcomes and factors such as GMFCS level [18], age at operation, and CP subtype. The assessments include measuring the ROM of ankle and knee using a manual goniometer. These measurements are conducted in a standardized position according to the CPUP manual (http://www.cpup.se), with the child and the goniometer arms positioned as specified. The primary endpoints of this study were the range of ankle dorsiflexion with knee extended and knee extension. Both were measured with the child in a supine position and hip extended.

Ankle and knee ROM data, both preoperatively and postoperatively, was extracted from the CPUP registry. Limits were defined as dorsiflexion of the ankle ≤ 0° or ≥ 20°, or knee extension < –10°. Children who underwent other surgical procedures on the lower limbs or where the type of gastrocsoleus lengthening could not be verified were excluded from the analysis. Additionally, children without reported ROM measurements before or after surgery were also excluded.

### Statistics

Baseline characteristics were summarized using descriptive statistics stratified by surgical group. Means, standard deviations (SD), and ranges summarize continuous variables, and frequencies and relative frequencies in percent summarize categorical variables. ROM outcomes were visualized through spaghetti plots and event outcomes via Kaplan–Meier curves.

The primary analysis of ankle ROM and secondary analysis of knee ROM were based on linear mixed-effects modeling, in part to accommodate the longitudinal data structure, to address the potentially irregular spacing of ROM measurements, and to account for missing outcome data. This method used a polynomial of time since surgery to model the trend of both individuals and population means over the study period (5 years before surgery to 10 years after), incorporating interactions to discern the effects of different types of surgery over time—0, 5, and 10 years after surgery. Model fit to data was evaluated by comparing nested models by Akaike’s Information Criterion (AIC) and likelihood ratio tests, as well as by plots of residual distributions. Both crude and adjusted models were produced.

For ROM analysis, all available measurements for the operated leg were included (5 years before to 10 years after surgery) in the analysis. For bilaterally operated children, measurements from the right side were included; measurements from the left side were omitted in order to reduce statistical modeling complexity.

Secondary survival endpoints, composite and component events, including both sides for bilaterally operated patients, were evaluated by means of Kaplan–Meier estimates of survival curves for each surgical group. Due to the clustered structure of the data, a likelihood ratio test based on a Cox shared gamma frailty model was used to test for any between-group differences, and pair-wise comparisons were performed using hazard rate ratios (HR) together with 95% confidence intervals (CI). All comparisons were made both as unadjusted, as well as adjusted for confounding covariates. Model fit to data was evaluated using Martingale and Schoenfeld residuals.

The set of covariates used for confounding adjustment was selected according to the modified disjunctive cause criterion [19]. Here, covariates that influence outcome and/or exposure, i.e., choice of surgery (age at surgery, the most recent ROM measurement preceding surgery [baseline ROM], GMFCS level, and CP subtype) were included in the final Cox models. In the mixed-effects models, ROM baseline was not included as it was already in the model as an outcome variable.

Sensitivity analyses were made to account for missing data and to gauge the effect of treating center as a potential confounder (see Supplementary data). No adjustment for multiplicity was performed in the current study, as it is observational and exploratory in nature [20]. Additional detail with regards to the statistical analysis and data management related to this project is given in the study plan published at ClinicalTrials.gov on March 13, 2023 (ID: NCT05783739).

### Ethics, use of AI tools, funding, and disclosures

Permission to extract data from the CPUP registry was obtained from the registry owner, Region Skane, and the study was approved by the Medical Research Ethics Committee at Lund University (LU-433-99). ChatGPT was used for language editing. The study was partially funded by Stiftelsen för bistånd åt rörelsehindrade i Skåne. The authors declare no conflict of interest. Complete disclosure of interest forms according to ICMJE are available on the article page, doi: 10.2340/17453674.2025.43387

## Results

**Figure 1:**
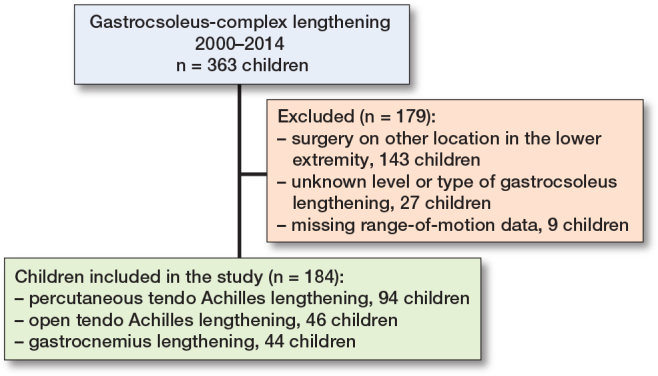
Inclusion and exclusion of children in the study.

363 children born 2000–2011 who underwent gastrocsoleus lengthening from 2000–2014 were identified. Of these, 143 had simultaneous operations on other locations, 9 had missing ROM data, and in 27 children the type of gastrocsoleus lengthening was unknown. These 179 children were excluded, resulting in 184 children who underwent isolated gastrocsoleus lengthening being included in the study (Figure 1). The number of ROM measurements per child varied according to their GMFCS levels, with a mean of 7.7 visits (SD 3.1, range 1–16). The mean follow-up time was 8.6 years (SD 2.7, range 4.2–14.1).

Most children underwent TAL, predominantly using a percutaneous approach. The age at surgery was similar across groups, and the majority of participants were classified as GMFCS level I or II and had either USCP or BSCP. A small proportion of children underwent a second gastrocsoleus lengthening during follow-up, with similar rates across surgical groups (Table 1).

**Table 1:** Number of children (%) according to sex, age at operation, age at reoperation, GMFCS-level, CP subtype, and type of operation unless otherwise specified

Factor	PTAL	OTAL	GCL	Total
Total number	94	46	44	184
Boys	55 (59)	26 (57)	25 (57)	106 (58)
Girls	39 (41)	20 (43)	19 (43)	78 (42)
Age at operation				
mean (SD)	6.8 (2.7)	6.7 (2.2)	6.7 (1.9)	6.7 (2.4)
range	3.0–14.0	2.2–12.0	3.4–10.8	2.2–14.0
Age at reoperation				
mean (SD)	9.9 (2.4)	9.3 (3.9)	9.6 (2.3)	9.5 (3.2)
range	6.4–18.8	6.9–12.5	7.3–14.0	6.4–14.0
Reoperations	14 (15)	4 (8.7)	7 (16)	25 (14)
GMFCS level				
I	53 (56)	28 (61)	16 (36)	97 (53)
II	17 (18)	10 (22)	15 (34)	42 (23)
III	3 (3.2)	4 (8.7)	4 (9.1)	11 (6.0)
IV	9 (10)	3 (6.5)	6 (14)	18 (10)
V	12 (13)	1 (2.2)	3 (6.8)	16 (8.7)
CP subtype				
USCP	43 (46)	22 (48)	15 (34)	80 (43)
BSCP	38 (40)	22 (48)	25 (57)	85 (46)
Dyskinetic CP	7 (7.5)	0 (0)	1 (2.3)	8 (4.4)
Ataxic CP	2 (2.1)	2 (4.4)	1 (2.3)	5 (2.7)
Spastic CP UNS	4 (4.3)	0 (0)	2 (4.6)	6 (3.3)

PTAL = percutaneous tendo Achilles lengthening, OTAL = open tendo Achilles lengthening, GCL = gastrocnemius lengthening, GMFCS = Gross Motor Function Classification System, CP = cerebral palsy, US = unilateral spastic, BS = bilateral spastic.

### Outcomes

**Figure 2:**
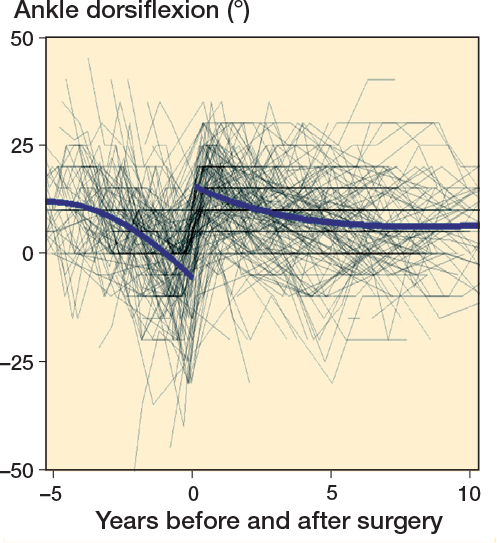
Individual measurements and population mean ankle dorsiflexion development before and after surgery.

Ankle ROM for the entire cohort declined to just below –5° preoperatively. Postoperatively, mean ankle ROM was about 15° and then gradually declined to about 5° at 10 years postoperatively (Figure 2, see Appendix). For patients with apparent positive ankle dorsiflexion (> 0°) prior to surgery, the mean time from last measurement to operation was 6.8 months (SD 4.4, range 2–19 months) (Table 2, see Appendix).

**Table 2:** Time from last preoperative examination to surgery and range of ankle dorsiflexion at last preoperative examination. Values are mean (standard deviation) and range

Factor	PTAL	OTAL	GCL	Total
Months to surgery, all				
mean (SD)	7.0 (4.9)	5.4 (4.2)	5.7 (4.7)	6.3 (4.7)
range	0.3–23.1	0.2–13.2	0.0–17.7	0.0–23.1
Months to surgery, sides with ankle dorsiflexion > 0°				
mean (SD)	7.4 (4.1)	7.7 (4.4)	5.4 (4.6)	6.8 (4.4)
range	1.2–18.6	0.8–13.2	0.0–12.9	2.0–18.6
Preoperative ankle dorsiflexion (°)				
mean (SD)	–3.4 (12.0)	–4.2 (13.2)	2.2 (9.6)	–2.2 (12.0)
range	–30 to 25	–40 to 20	–25 to 30	–40 to 30

For abbreviations, see Table 1.

**Figure 3:**
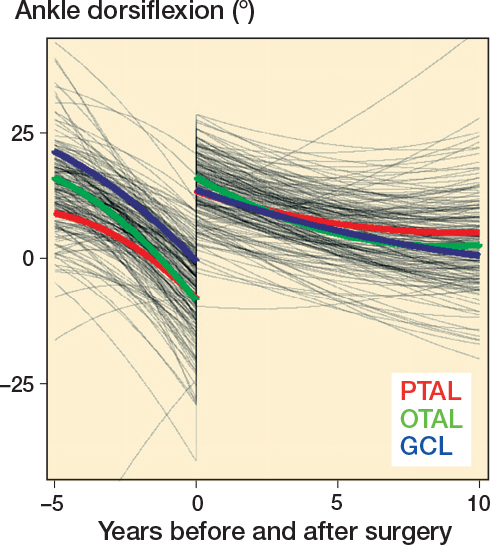
Mixed effects modeling for mean ankle dorsiflexion before and after surgery. PTAL = percutaneous tendo Achilles lengthening, OTAL = open tendo Achilles lengthening, GCL = gastrocnemius lengthening.

Regarding ankle ROM development, children with PTAL and OTAL had a preoperative mean dorsiflexion of almost –10°, and those with GCL around 0°. Postoperatively, the mean ankle dorsiflexion was about 15° in all 3 groups followed by a gradual decline to between 0° and 5° at 10-year follow-up (Figure 3). The differences between the 3 groups were small with CIs excluding any differences larger than 10.4° either preoperatively, postoperatively, at 5 years’, or at 10 years’ follow-up (Table 3).

**Table 3:** Unadjusted and adjusted differences of mean ankle dorsiflexion (°) just postoperatively, at 5 years’ and at 10 years’ follow-up (FU) between each pair of treatment groups

Follow-up Comparison	Unadjusted Mean difference (CI)	Adjusted ^a^ Mean difference (CI)
Postoperatively		
OTAL vs PTAL	2.8 (–0.65 to 6.2)	2.9 (–0.54 to 6.4)
GSL vs PTAL	0.09 (–3.6 to 3.7)	–0.13 (–3.6 to 3.8)
GSL vs OTAL	–2.7 (–6.8 to 1.5)	–2.8 (–7.0 to 1.4)
At 5 years’ follow-up		
OTAL vs PTAL	–2.0 (–4.8 to 0.77)	–1.8 (–4.6 to 0.96)
GSL vs PTAL	–1.5 (–4.6 to 1.7)	–1.4 (–4.5 to 1.7)
GSL vs OTAL	0.55 (–3.0 to 4.1)	0.42 (–3.1 to 3.9)
At 10 years’ follow-up		
OTAL vs PTAL	–2.5 (–7.6 to 2.7)	–2.3 (–7.4 to 2.7)
GSL vs PTAL	–4.6 (–11 to 1.5)	–4.4 (–10 to 1.5)
GSL vs OTAL	–2.1 (–8.7 to 4.4)	–2.1 (–8.5 to 4.4)

For abbreviations, see Table 1 and CI = 95% confidence interval.

a Adjusted for age at surgery, the most recent ROM measurement preceding surgery (baseline ROM), GMFCS level, and CP subtype.

**Figure 4:**
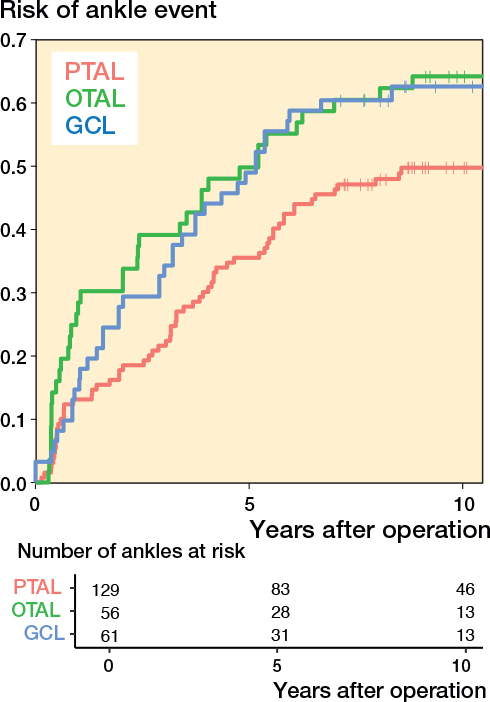
Risk of having experienced the event defined as ankle dorsiflexion ≤ 0° or ≥ 20° as a function of time after operation. For abbreviations, see Figure 3.

Survival curves showed a lower event risk in children who underwent PTAL compared with those who underwent OTAL or GCL. The difference was statistically significant in both the unadjusted likelihood ratio test (P = 0.003), and the adjusted analysis (P = 0.001) (Figure 4).

**Figure 5:**
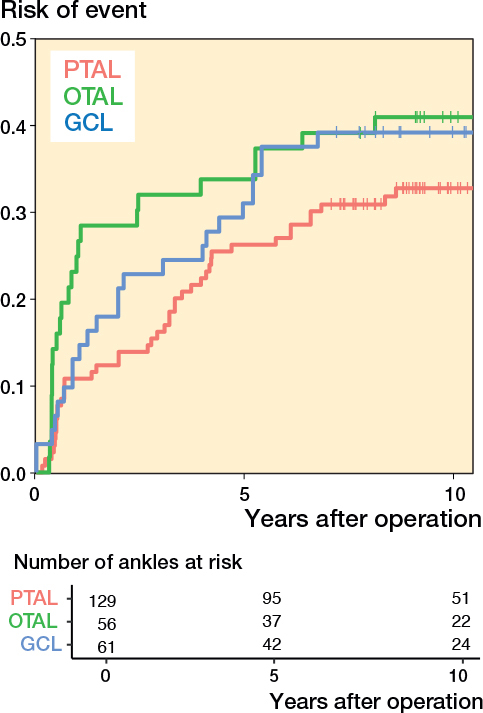
Risk of having experienced the event defined as ankle dorsiflexion ≥ 20° as a function of time after operation. For abbreviations, see Figure 3.

**Figure 6:**
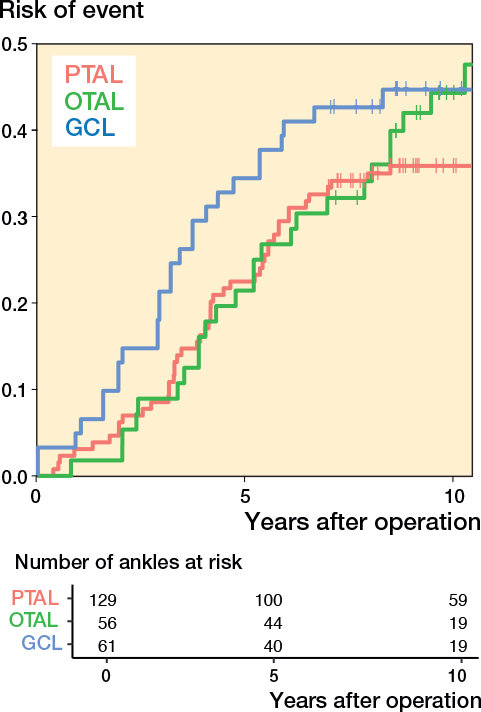
Risk of having experienced the event defined as ankle dorsiflexion ≤ 0° as a function of time after operation. For abbreviations, see Figure 3

The unadjusted likelihood ratio test showed statistically significant difference among the 3 surgery groups when event was defined as only dorsiflexion of the ankle > 20°, P = 0.004 (PTAL lower event rate than OTAL and OTAL lower than GCL) (Figure 5, see Appendix). When event was defined as only dorsiflexion of the ankle < 0°, the intended statistical method could not be used, likely due to a small number of events (Figure 6, see Appendix).

PTAL showed a lower event rate compared with OTAL (adjusted HR 2.5, CI 1.1–5.7, P = 0.03) (Table 4). PTAL was also observed to have a lower event rate compared with GCL (adjusted HR 2.0, CI 0.85–4.6, P = 0.1); however, the difference was not statistically significant (Table 4). Results regarding confounding adjustment variables—age at surgery, GMFCS level, preoperative ROM, and CP subtypes—were uncertain, as indicated by wide confidence intervals. None exhibited a statistically significant association with the outcome (Table 5, see Appendix).

**Table 4:** Hazard rate ratios (HR) comparing event rates between surgery methods, estimated using a Cox shared gamma frailty model, including data on both sides on bilaterally operated on patients

Comparison	Crude (n = 246)		Adjusted (n = 211)	
	HR (CI)	P value	HR (CI)	P value
Ankle events				
OTAL vs PTAL	2.3 (1.1–4.7)	0.02	2.5 (1.1–5.7)	0.03
GCL vs PTAL	2.0 (1.01–4.1)	0.05	2.0 (0.85–4.6)	0.1
Knee events ^a^				
OTAL vs PTAL	0.5 (0.24–1.1)	0.1	0.7 (0.29–1.4)	0.3
GCL vs PTAL	1.1 (0.6–2.0)	0.8	1.1 (0.57–2.1)	0.8

Hazard rate ratios adjusted for baseline ROM, GMFCS, and CP subtype. Event limit for ankle defined as dorsiflexion ≤ 0° or ≥ 20° and for knee as extension ≤ –10°.

For abbreviations, see Table 1 and CI = 95% confidence interval.

a Results produced using Cox regression with cluster robust standard errors, as opposed to the Cox shared gamma frailty model used for remaining results.

**Table 5:** Hazard rate ratios (HR) comparing event rates between surgery methods together with HRs for the adjustment variables, estimated using a Cox proportional hazards model with cluster robust standard errors, including data on both sides on bilaterally operated patients. Here the event is defined as ankle dorsiflexion ≤ 0° or ≥ 20°

Comparison	Crude (n = 246)		Adjusted (n = 211)	
	HR (CI)	P value	HR (CI)	P value
OTAL vs PTAL	2.3 (1.1–4.7)	0.02	2.5 (1.1–5.7)	0.03
GCL vs PTAL	2.0 (1.01–4.1)	0.05	2.0 (0.85–4.6)	0.1
Ankle dorsiflexion preoperatively			1.0 (0.99–1.04)	0.2
GMFCS I–III vs IV–V			0.70 (0.26–1.9)	0.5
Age at operation			0.95 (0.83–1.1)	0.5
Ataxic CP vs USCP			0.34 (0.06–2.0)	0.2
BSCP vs USCP			0.65 (0.31–1.4)	0.3
Dyskinetic CP vs USCP			0.34 (0.05–2.1)	0.2
CP UNS vs USCP			0.34 (0.04–2.9)	0.3

For abbreviations, see Table 1 and CI = 95% confidence interval, UNS = unspecified diagnosis.

The number of legs with knee extension ≤ –10° increased from 24 preoperatively to 74 at the last follow-up, with 42 of these legs exhibiting knee extension ≤ –20° (Table 6, Appendix).

**Table 6:** Number of operated legs with knee contracture preoperatively and at last follow-up in different GMFCS-levels and CP subtypes (n = 246)

Factor	Knee extension ≤ –10° preoperatively	Knee extension ≤ –10° at last follow-up	Knee extension ≤ –20° at last follow-up
GMFCS level			
I	3	8	1
II	3	12	5
III	3	10	4
IV	6	17	8
V	9	27	24
CP subtype			
USCP	3	8	2
BSCP	15	43	23
Dyskinetic CP	2	12	10
Ataxic CP	1	5	2
Spastic CP UNS	3	6	5
Total	24	74	42

For abbreviations, see Table 1 and UNS = unspecified diagnosis.

**Figure 7:**
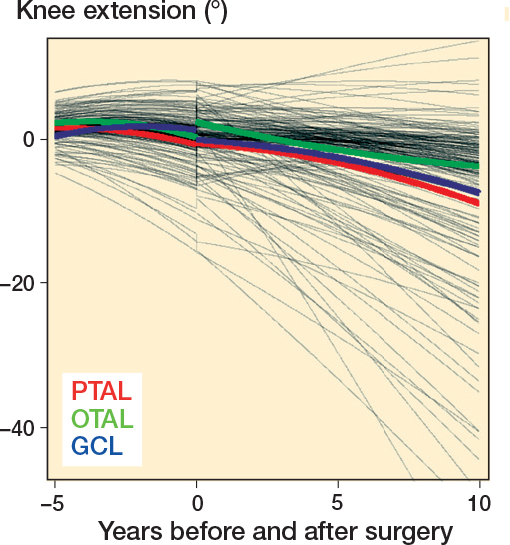
Development of individual and population mean knee extension before and after surgery produced using mixed-effects regression modeling. For abbreviations, see Figure 3.

**Figure 8:**
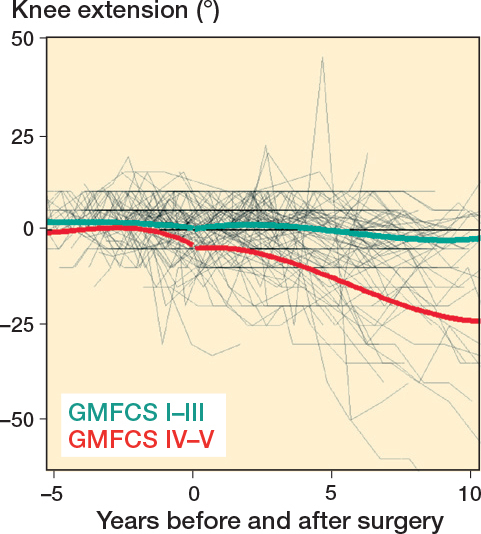
Individual knee extension development and means for GMFCS IV–V and GMFCS I–III.

Mean preoperative knee extension in the whole cohort was just below 5°. Postoperatively, mean knee extension declined in all surgery groups, with the most pronounced decline observed in the PTAL and GCL groups (Figure 7). Regarding knee ROM development, the differences between the 3 groups were small even if OTAL was associated with slightly greater knee extension during follow up, compared with PTAL (Table 7). The decline in knee extension was also more pronounced in GMFCS IV–V, around –25°, compared with about –5° in GMFCS I–III (Figure 8, see Appendix).

**Table 7:** Unadjusted and adjusted differences of mean knee extension just postoperatively, at 5 years’ and at 10 years’ follow-up (FU) between each pair of treatment groups

Follow-up Comparison	Unadjusted Mean difference (CI)	Adjusted ^a^ Mean difference (CI)
Postoperatively		
OTAL vs PTAL	2.9 (0.75 to 5.1)	2.5 (0.40 to 4.7)
GCL vs PTAL	0.43 (–1.9 to 2.8)	0.44 (–1.8 to 2.7)
GCL vs OTAL	–2.5 (–5.1 to 0.13)	–2.1 (–4.6 to 0.46)
At 5 years’ follow-up		
OTAL vs PTAL	1.8 (–0.72 to 4.3)	1.6 (–0.81–to 4.0)
GCL vs PTAL	0.79 (–2.2 to 3.8)	0.98 (–1.8 to 3.7)
GCL vs OTAL	–1.0 (–4.3 to 2.3)	–0.59 (–3.7 to 2.5)
At 10 years’ follow-up		
OTAL vs PTAL	5.2 (0.40 to 10)	5.1 (0.43 to 9.8)
GCL vs PTAL	1.5 (–4.2 to 7.2)	1.9 (–3.6 to 7.5)
GCL vs OTAL	–3.7 (–9.9 to 2.6)	–3.2 (–9.3 to 2.9)

For abbreviations, see Table 1 and CI = 95% confidence interval.

a Adjusted for age at surgery, the most recent ROM measurement preceding surgery (baseline ROM), GMFCS, level and CP sub-type.

**Figure 9:**
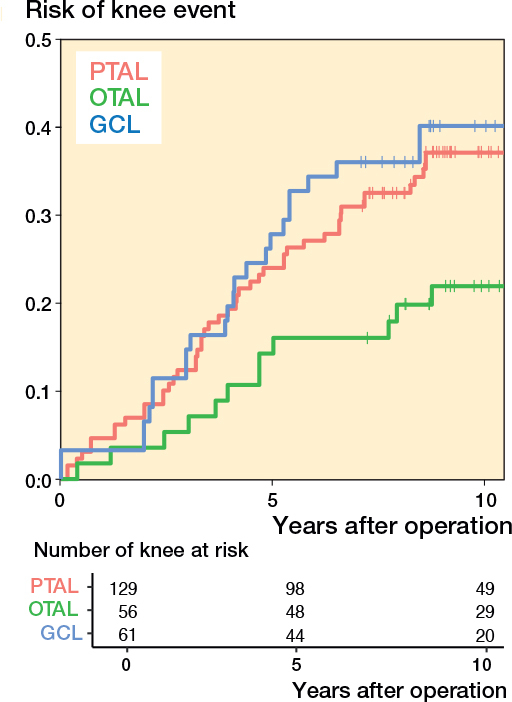
Risk of having experienced the event defined as knee extension ≤ –10° as a function of time after operation. For abbreviations, see Figure 3.

Survival estimates for events related to knee ROM indicated that children who underwent OTAL had a lower event rate compared with those who underwent PTAL and GCL (Figure 9). Due to low event rates, differences could not be tested using the intended statistical method. Instead, a Cox regression model with cluster-robust standard errors was used. In both unadjusted and adjusted analyses (adjusted for GMFCS and CP subtype only), no statistically significant differences in knee extension were identified between OTAL, GCL, and PTAL (Table 8, see Appendix). Furthermore, the wide confidence intervals indicate low statistical precision. Due to the insufficient number of events, a fully adjusted model was not supported.

**Table 8:** Hazard rate ratios (HR) comparing event rates between surgery methods together with HRs for the adjustment variables, estimated using a Cox proportional hazards model with cluster robust standard errors, including data on both sides on bilaterally operated patients. Here the event is defined as knee extension ≤ –10°

Comparison	Crude (n = 246)		Adjusted (n = 243)	
	HR (CI)	P value	HR (CI)	P value
OTAL vs PTAL	0.52 (0.24–1.1)	0.1	0.65 (0.29–1.45)	0.3
GCL vs PTAL	1.1 (0.60–2.0)	0.8	1.1 (0.57–2.1)	0.8
GMFCS I–III vs IV– V			0.56 (0.28–1.1)	0.1
Age at operation			1.0 (0.89–1.2)	0.7
Ataxic CP vs USCP			2.3 (0.65–7.9)	0.2
BSCP vs USCP			1.8 (0.88–3.6)	0.1
Dyskinetic CP vs USCP			3.6 (1.3–10)	0.01
CP UNS vs USCP			4.7 (1.3–17)	0.02

For abbreviations, see Table 1 and CI = 95% confidence interval, UNS = unspecified diagnosis.

Sensitivity analyses revealed only minor numerical differences in results when accounting for treating center or missing data, indicating there was no need to account for these issues.

## Discussion

The aim of our study was to analyze the development of ankle and knee ROM after isolated PTAL, OTAL, and GCL. The main finding was that mean ankle and knee ROM development was similar among the 3 surgery groups. The largest possible difference between the groups, based on CIs, was approximately 5° at 5 years. This is close to the reported goniometer measurement error [21,22] and not deemed clinically relevant. At 10 years the confidence intervals were wider due to fewer measurements, with no clinically relevant differences. The event rate for ankle ROM was statistically significantly lower after PTAL compared with OTAL. It was also lower for PTAL compared with GCL, although the difference was not statistically significant. For knee extension, results were associated with considerable statistical uncertainty, and no firm conclusions could be drawn.

The outcome variables included knee extension and ankle dorsiflexion with extended knee, as these are the joints primarily affected by gastrocsoleus surgery. In the typically developing population, ankle dorsiflexion ranges between 10° and 20° but there is limited consensus on what constitutes a clinically relevant ankle equinus contracture or the indication for surgery, with reported values ranging from –10° to 10°. Cut-off values were selected based on these ranges and normal ankle ROM [6,23-25]. The reason for using a composite variable (ROM ≤ 0° or ≥ 20°) was the lack of statistical power for separate analyses (even if presented in Figures 5 and 6, see Appendix) and the argument that children within this range probably would not need repeat surgery. Knee hyperextension of 5–10° is considered normal and knee extension of < –10° has been described as relevant knee contracture [24,26].

The pooled risk of recurrence (reoperation) after TAL was 15% in a recent meta-analysis [27], which is comparable to our finding of 14% risk of recurrence, although our estimate does not account for individual differences in follow-up time.

We found no statistically significant differences in event rates related to age at surgery, preoperative ROM, and CP subtype. This may be due to lack of statistical precision, indicated by large confidence intervals due to limited sample size, as the study was not designed for this purpose. With respect to these factors, earlier publication results vary. For example, young age at primary surgery has been reported to be a risk factor for reoperation in some studies [8,11,28,29], while others found no such association [12,30]. A high degree of preoperative equinus has also been found to be a risk factor for reoperation [6,12,13], while others found no statistically significant association [8,27]. USCP has been related to recurrent equinus and BSCP has been described to increase the risk of over-correction [6,31]. On the other hand, Chung et al. found no statistically significant association between CP subtype and the risk of reoperation [12]. Kero et al. reported on a large cohort (15-year follow-up of 319 children operated on with only PTAL and OTAL), but calcaneal gait was described in only 3.5% of the children and PTAL was preferred for its simplicity and lower level of wound complications [29]. In a recent meta-analysis, Horsch et al. [27] pointed out the low level of evidence in available studies. A major weakness of the included studies was their failure to investigate and adjust for confounding bias. Additionally, as most studies present only P values, it is unclear whether the results are conflicting or if the variation is a consequence of a lack of statistical precision and power. If the studies had included measures of effect and corresponding CIs, these issues could have been more formally evaluated. This highlights the necessity for well-designed, large studies, and proper reporting [27].

There was no clinically relevant difference in mean ankle ROM development between the 3 treatment groups. This is contrary to the general opinion that children with BSCP should not be treated with surgical lengthening at the tendinous level due to the risk of overlengthening and subsequent crouch [9]. Although ankle ROM is not a full measure of functional outcome, and decisions regarding gastrocsoleus lengthening should be based on a comprehensive individual assessment where passive ROM development is only one of many factors to consider, these findings are important and warrant consideration.

Interestingly, the development of knee ROM over time in our study followed a similar pattern to that observed in the total population of children with CP in Sweden [32]. In this study by Nordmark et al., mean knee extension decreased by 6° between the ages of 2 and 14 years, which is consistent with the decrease observed over 10 years in our study. These findings suggest that knee ROM development in children with CP is largely independent of the type of surgical intervention on the gastrocsoleus complex.

We used two different statistical approaches, mixed-effect regression models and survival analysis, yielding seemingly different results. Mixed-effect models showed only minor differences between the surgical methods, while survival analysis revealed statistically significant differences. This could be explained by the fact that they analyze different outcomes. The former focuses on the mean ROM and the latter on the event that ROM reaches a specific threshold. Thus, while the mean ROM is similar between groups, another aspect of the outcome distribution differs and is reflected by the survival analysis. These findings highlight that different statistical methods can yield varying results, emphasizing the need for cautious interpretation and diverse analytical approaches when evaluating these types of studies.

### Limitations

A limitation in this study is the lack of patient-related outcomes such as functional gait analysis. It would have made it possible to draw more conclusions regarding choice of surgical method. Another limitation is that ankle ROM includes only measurements with extended knee, making it impossible to isolate the soleus component. Access to surgical records (but not full patient records) did not include direct preoperative clinical examination and it is therefore not possible to evaluate whether the appropriate surgical approach was chosen. A further limitation is the varying number of follow-up examinations and intervals. While this was adjusted for in the mixed-effect model, it could have biased results from the Cox regression model. However, adjusting for GMFCS level, which partly accounts for differences in follow-up patterns, should have reduced this bias. The variation in time from last assessment to operation and in follow-up intervals could also affect our ability to adjust for baseline ROM measurements, as it introduced variation in the measurement time points relative to baseline.

### Strengths

The strength of this study is the inclusion of a large cohort of children with CP, representing the entire population of those who underwent isolated gastrocsoleus lengthening in Sweden during the follow-up period and the substantial number of follow-up measurements. This study comprehensively describes the longitudinal development of knee and ankle ROM following different surgical procedures to lengthen the gastrocsoleus complex.

### Conclusion

We showed that the development of mean ankle and knee ROM after isolated PTAL, OTAL, and GCL exhibited similar patterns. Those who underwent isolated PTAL had a lower rate of events than OTAL or GCL with respect to ankle ROM.

## Notes

Study design: OL, GH, PW, HLP. Data collection: OL, GH. Statistical analysis: PW, OL. Manuscript preparation: OL, GH, PW, HLP.

Handling co-editor: Ilkka Helenius

*Acta* thanks Julia F. Funk, Unni Narayanan, Terje Terjesen, and an anonymous reviewer for help with peer review of this manuscript.

## Appendix

### Statistical methods – continued

Sensitivity analyses were made to gauge the effect of treating center as a potential confounder, by including it as a frailty in the frailty model and a random effect in the mixed effects model. To simplify the frailty model, one leg was omitted in bilateral cases. Additional sensitivity analyses to account for missing data using multiple imputation methods in the MICE package in R [33] was performed for any analysis exceeding 5% missing data. Here, the same variables were in the imputation model as in the analysis model.
